# Group Norms Influence Children’s Expectations About Status Based on Wealth and Popularity

**DOI:** 10.3389/fpsyg.2022.816205

**Published:** 2022-05-11

**Authors:** Kathryn M. Yee, Jacquelyn Glidden, Melanie Killen

**Affiliations:** Department of Human Development and Quantitative Methodology, University of Maryland, College Park, MD, United States

**Keywords:** group norms, status, wealth, popularity, intergroup

## Abstract

Children’s understanding of status and group norms influence their expectations about social encounters. However, status is multidimensional and children may perceive status stratification (i.e., high- and low-status) differently across multiple status dimensions (i.e., wealth and popularity). The current study investigated the effect of status level and norms on children’s expectations about intergroup affiliation in wealth and popularity contexts. Participants (*N* = 165; age range: 5–10 years; *M*_*age*_ = 7.72 years) were randomly assigned to hear two scenarios where a high- or low-status target affiliated with opposite-status groups based on either wealth or popularity. In one scenario, the group expressed an inclusive norm. In the other scenario, the group expressed an exclusive norm. For each scenario, children made predictions about children’s expectations for a target to acquire social resources. Novel findings indicated that children associated wealth status to some extent, but they drew stronger inferences from the wealth dimension than from the popularity dimension. In contrast to previous evidence that children distinguish between high- and low-status groups, we did not find evidence to support this in the context of the current study. In addition, norms of exclusion diminished children’s expectations for acquiring social resources from wealth and popularity groups but this effect was more pronounced between wealth groups. We found age differences in children’s expectations in regards to norms, but not in regards to status. The implications of how these effects, in addition to lack of effects, bear on children’s expectations about acquiring resources are discussed.

## Introduction

Social status reflects the level of prestige and deference that an individual or group is afforded by others (Anderson et al., 2015). Status stratification is prevalent across societies and young children attend to status cues based on dimensions such as power, wealth, dominance, and social acceptance. By their preschool years, children accurately identify individuals who are high- or low-status, which further guides their expectations about others’ traits, abilities, and behavior (Brey and Shutts, 2015; Charafeddine et al., 2015; Shutts et al., 2016; Gülgöz and Gelman, 2017; Enright et al., 2020). Often, children associate multiple dimensions of status. For example, they view wealthy targets as popular (Shutts et al., 2016) and associate physical dominance with competence and possessing more resources (Charafeddine et al., 2015). Moreover, studies examining various status dimensions find that children associate more positive attributes with high-status individuals and exhibit stronger preferences for them than for low-status individuals (Horn, 2006; Newheiser et al., 2014; Mistry et al., 2015; Shutts et al., 2016; Enright et al., 2020).

Children may favor high-status peers over low-status peers for a variety of reasons. In addition to inferring that individuals possess similar rank across status dimensions, they may broadly infer positive traits from positive status information (Cain et al., 1997). For example, children associate the wealthy with more positive traits (e.g., smart, hardworking, clean, good, honest, polite) than the poor (Mistry et al., 2015). They may also infer positive traits in order to justify existing disparities observed between status groups (Baron and Banaji, 2009; Newheiser et al., 2014).

Alternatively, children may be motivated to identify with groups that are positively distinguished in order to enhance their own self-esteem (Abrams and Rutland, 2008; Nesdale, 2008). Status distinctions may indicate to children the extent to which an individual can functionally benefit others. Affiliation with popular peers, for instance, can enhance one’s own social standing (Dijkstra et al., 2010). Children expect wealthy peers to share more resources than non-wealthy peers (Ahl and Dunham, 2019; Ahl et al., 2019) and also allocate more resources to peers who they expect to share with them and help them (Dunham et al., 2011; Renno and Shutts, 2015).

Despite associations between multiple dimensions of status, no studies to date have compared children’s expectations about the benefits of cross-status affiliation between different dimensions. Moreover, although children expect to receive material resources from the wealthy, less is known about whether children also expect to receive relatively more social benefits from wealthy peers than non-wealthy peers. The current study first aims to investigate children’s associations between two dimensions of status: wealth and popularity. In addition, we aim to extend previous literature by comparing children’s expectations about acquiring social benefits through cross-status affiliation in wealth and popularity contexts.

### Conceptions of Wealth Status

Children are aware of wealth stratification from a young age and often favor wealthy peers over non-wealthy peers. Children view wealthy individuals as more competent (Woods et al., 2005; Sigelman, 2012; Li et al., 2014; Mistry et al., 2015; Shutts et al., 2016), more likely to share (Ahl and Dunham, 2019; Ahl et al., 2019), and having more friends (Shutts et al., 2016) than non-wealthy individuals. Moreover, children as young as 4 years of age explicitly and implicitly prefer wealthy peers over non-wealthy peers (Olson et al., 2012; Horwitz et al., 2014; Li et al., 2014; Newheiser et al., 2014; Shutts et al., 2016).

Despite these biases, children are simultaneously sensitive to the needs of the economically disadvantaged. They view poverty as unfair and recognize that the poor lack basic necessities as well as a social network (Chafel and Neitzel, 2005). Children increasingly attempt to reduce inequality by allocating more resources and opportunities to low-wealth peers than high-wealth peers with age (Li et al., 2014; Elenbaas and Killen, 2019; Zhang et al., 2021). In contrast to 4-year-olds, 8-year-olds reported more negative emotions after hypothetically excluding an economically disadvantaged peer (Dys et al., 2019). However, some evidence suggests that after age 11, children increasingly legitimize wealth inequality and their beliefs that the rich should give to the poor then decline (Leahy, 1990).

Children’s preferences for the wealthy appear to be at odds with their egalitarian beliefs. Li et al. (2014) found that 4- and 5-year-olds preferred to be friends with a resource rich target than a resource poor target, but allocated more toys to the resource poor target. Interestingly, when children forgot which target initially possessed more resources due to a delay between the preference and allocation tasks, they favored the resource rich target in both their preferences and allocations. Thus, children’s wealth preferences may be driven by automatic and unconscious positive associations. Moreover, their attitudes and behavior may not consistently favor the wealthy when moral concerns arise.

Studies focusing on children’s trait associations with wealth groups, suggest that children’s preferences may be particularly driven by beliefs that wealthy individuals are competent and likely to share (Woods et al., 2005; Sigelman, 2012; Li et al., 2014; Mistry et al., 2015; Shutts et al., 2016; Ahl and Dunham, 2019; Ahl et al., 2019). At the same time, children as young as 8 years view wealthy individuals as greedy, selfish, and exclusive (Elenbaas and Killen, 2019; Burkholder et al., 2020). Preferences for the wealthy may not merely be driven by beliefs that they are particularly likeable. Rather, affiliating with individuals who are viewed as competent and able to share their resources may provide certain economic and social benefits that children find attractive.

### Conceptions of Popularity Status

Peer popularity is another important dimension of status for children and is defined as individual’s prestige, visibility, and reputation among peers (Cillessen and Marks, 2011). Traditional sociometric methods (for review, see Cillessen, 2009) have assessed popularity using peer nomination procedures, where children rank their peers by who they like the most to the least. Those who received the most nominations were then classified as popular and those with the least were classified as unpopular. However, peer relation studies now distinguish popularity from mere peer preference. For example, a study of 9- to 13-year-olds found that children who were explicitly nominated as the most popular exhibited more social dominance (i.e., ability to compete for or control material and social resources) than those who were nominated as the most well-liked (Lease et al., 2002). The same study also found an association between popularity and wealth in terms of having money to spend and high-quality possessions such as expensive clothing and a very nice house. Younger children in grades 3–5 also identify popular peers as those who influence others’ behavior and set social norms (Lease et al., 2020). However, peers who were considered both popular and well-liked were distinguished from the broader popular group by prosocial qualities and being less likely to use ridicule or model misbehavior in order to influence others. Thus, popular peers are viewed as both prosocial and antisocial (LaFontana and Cillessen, 2002).

Children’s associations between popularity and peer preference decline between early childhood and adolescence (Cillessen and Marks, 2011). This may be due, in part, to children’s increasing consideration of group dynamics (e.g., status hierarchies, norms, and distinctions between personal and consensus-based judgments). There is also evidence that popularity becomes increasingly related to antisocial behavior such as aggression (Sandstrom, 2011). In addition, children increasingly prioritize popularity status. Compared to children in grades 1–4, children in grades 5–8 were more likely to make decisions that increase or maintain their popularity status at the expense of friendship, compassion, achievement, and rule adherence (LaFontana and Cillessen, 2010).

In addition, children may be more willing to disregard or admire a high-status peer’s antisocial behavior than a low-status peer’s. Children explicitly prefer popular peers over unpopular peers even if they hold implicit negative attitudes toward them (Lansu et al., 2012), and choose to include them in activities over unpopular peers (Horn, 2006). While prosocial behavior predicted higher perceived friendship quality among unpopular children, popular children were viewed as possessing high quality friendships regardless of their prosocial tendencies (Poorthuis et al., 2012). Even in the absence of prosocial traits, popular peers may possess other redeeming qualities such as being powerful and influential, which may help others enhance their social standing (Cillessen and Marks, 2011). A study among adolescents found that an individual’s popularity and likeability increased the closer they affiliated with popular peers (Dijkstra et al., 2010). However, it’s unclear whether elementary school-aged children view affiliation with popular peers as a means for achieving status or acquiring additional social resources.

### Group Norms and Status

Children’s understanding of social norms can powerfully regulate their intergroup attitudes and behavior (Nesdale et al., 2005; Rutland et al., 2005; Bennett, 2014; McGuire et al., 2017). Social norms promote group functioning by establishing a sense of common ground and by regulating within-group behavior (Feldman, 1984; Abrams et al., 2003a,b). The manifestation of prejudice and discrimination depends on the strength of one’s group identification, perceptions of threat and competition, and the extent to they view these attitudes and behaviors as in line with group standards (Rutland and Killen, 2015). For instance, children who were assigned to a group with a norm of exclusion favored their own group and expressed attitudes that were consistent with their group’s norm (Nesdale et al., 2008). Under some circumstances, norms can also moderate children’s biases toward their own group. When children view an outgroup as holding a competitive or exclusive norm, they are more likely to dislike and lack empathy for outgroup members than when the outgroup is perceived to be cooperative or inclusive (Nesdale et al., 2005, 2007; Nesdale and Dalton, 2010). However, children are inclined to view their own group’s positively and therefore, may be more likely to view their own group as more inclusive than an outgroup when norms are not explicit. For example, Non-Arab American adolescents expected their own group to include peers based on shared interests, but expected Arab American peers to include peers based on ethnicity (Hitti and Killen, 2015). Whether they show out-group prejudice or not will depend in part on the strength of their identification with their group, how much they feel their group is being threatened, and if they understand and believe that showing such prejudice is consistent with the expectation of their group (i.e., the in-group norm).

Further, the way in which norms guide children’s behavior depends on group status. In a study where participants were assigned to an advantaged or disadvantaged group that held either a norm of equality or equity, disadvantaged adolescents allocated more resources to their in-group when their group held a norm of equity, rather than equality (McGuire et al., 2019). In contrast, advantaged adolescents distributed resources equally even when their group prescribed an equity norm. Group norms are based on a consensus among peers. However, individuals who possess substantial social status have greater influence over the attitudes and behaviors of others. For example, popular children have the ability to exert control over group norms by serving as visible models of group standards and reinforcing norms through their social networks (Sandstrom, 2011). While wealthy children vary in their visibility and social connectedness, they may have the ability to influence others due to their control over material resources (Ahl and Dunham, 2019; Ahl et al., 2019). Thus, norms may be more strongly determined by high status groups and they may impact status groups differently.

Children’s understanding of group dynamics becomes increasingly sophisticated with age (Nesdale et al., 2005; Abrams and Rutland, 2008; Abrams et al., 2009; Rutland et al., 2010). For example, a study by McGuire et al. (2019) found differences in how children considered their group’s relative social standing and group norms when deciding how to allocate resources. Adolescents allocated more resources to their disadvantaged in-group over a disadvantaged outgroup when their ingroup held a norm of equity. In contrast, children prioritized equal allocations regardless of the norm and even when it perpetuated their own disadvantage. Studies that investigate children’s reasoning further shed light on changes in their cognition. For instance, older children are more likely to prioritize group loyalty (Rutland and Killen, 2015) and cite concerns about group functioning in order to justify exclusion than younger children (Hitti and Mulvey, 2021). This increasing awareness of competing factors contributes to a shift in children’s motivations and behavior during intergroup encounters.

### The Current Study

The first goal of this study was to investigate children’s associations between wealth and popularity status.

**H1:** We expected that participants in the current sample would demonstrate a bidirectional association between wealth and popularity status, such that they would view wealthy targets as more popular than non-wealthy targets and would view popular targets as wealthier than unpopular targets. Investigating these associations served to clarify existing literature about the relationship between wealth and popularity. Despite some evidence that children conflate features of wealth and popularity (Lease et al., 2002; Charafeddine et al., 2015; Shutts et al., 2016; Gülgöz and Gelman, 2017; Enright et al., 2020), studies have not compared the relative strength of inferences across these two dimensions.

Our second goal was to investigate and compare children’s expectations about acquiring social resources through cross-status affiliation in wealth and popularity contexts. Specifically, we examined children’s expectations about positive group attitudes toward a cross-status target, the target’s personal enjoyment from cross-status affiliation, and the group’s future inclusion of the target. The interplay between group norms and social status was a primary focus of our investigation and we predicted that several factors would contribute to children’s expectations for social resources.

**H2:** We predicted that overall, participants would have higher expectations for a target to acquire social resources from a group that held a norm of inclusion rather than exclusion, but that the extent to which the norm influenced expectations would depend on the group’s status level. Children’s expectations about others’ attitudes and behavior are sensitive to their perceptions of how individuals conform or deviate from group standards (Rutland and Killen, 2015). Exclusive norms can exacerbate in-group biases and facilitate prejudice, while inclusive norms can elicit positive intergroup attitudes and have been shown to mitigate prejudice toward low-status groups (Nesdale et al., 2007; Nesdale and Lawson, 2011). We anticipated that children would also have higher expectations for a target to acquire social resources through affiliation with a high-status group than a low-status group. Children expect to receive material resources from wealthy peers (Ahl and Dunham, 2019; Ahl et al., 2019) and to increase their social network from popular peers (Dijkstra et al., 2010). If wealth and popularity status are associated, children may expect there to be social benefits to affiliation with the wealthy as well. These expectations may contribute to children’s preferences for high-status groups, which have been well-documented (Horn, 2006; Newheiser et al., 2014; Mistry et al., 2015; Shutts et al., 2016; Enright et al., 2020). As a result, children might have higher expectations for acquiring social resources from a high-status group than a low-status group, even when both groups have a norm of inclusion. Further, children may also be willing to overlook antisocial attributes of peers when they have redeeming qualities such as high-status (Cillessen and Marks, 2011; Poorthuis et al., 2012). Compared to an inclusive low-status group, for instance, children may still have relatively high expectations for an individual to acquire social resources from an exclusive high-status group. Alternatively, children might have relatively low expectations for acquiring resources from an exclusive high-status group. Children view high-status peers as setting norms (Gülgöz and Gelman, 2017; Lease et al., 2020) so a norm of exclusion could be viewed as a more difficult barrier to overcome with a high-status group. In addition, a high-status group might ultimately reject a low-status individual because affiliation with them could be viewed as a threat to their group’s positive social standing (Nesdale et al., 2005). They may also view high-status group as particularly exclusive even when one member is inclusive (Lease et al., 2002; Cillessen and Marks, 2011; Elenbaas and Killen, 2019; Burkholder et al., 2020).

**H3:** We also expected the effect of norm on children’s expectations for acquiring resources to be more pronounced when affiliation occurs between wealth groups than between popularity groups. Wealth distinctions may be more salient to children than popularity distinctions. Children view the wealthy as competent and hardworking, while the view the poor as incompetent and lazy (Woods et al., 2005; Sigelman, 2012; Li et al., 2014; Mistry et al., 2015; Shutts et al., 2016). Some children are also more favorable to the poor and distinguish the wealthy as selfish and entitled, while the poor are viewed as generous (Elenbaas and Killen, 2019; Burkholder et al., 2020). Evidence that children readily endorse stereotypes about high- and low-wealth groups suggests that wealth is a particularly informative status distinction. Moreover, children expect their peers to preferentially include others on the basis of wealth due to more perceived comfort with their own group (Burkholder et al., 2021). They may assume that groups are exclusive even in the absence of an explicit norm (Burkholder et al., 2020, 2021) and thus, more readily generalize an individual group member’s exclusive preferences to a wealth group than a popularity group. On its own, popularity status may be less informative for predicting behavior during childhood. Children may be less inclined to generalize an exclusive preference to a popularity group since there’s no evidence that they stereotype popularity groups as particularly exclusive or negative toward each other before adolescence. Rather, they may expect more variability among the members of popularity groups some group members more readily than they do among wealth groups. For example, they recognize that some popular individuals are more well-liked by their peers than others and that popular individuals exhibit both prosocial and antisocial qualities (LaFontana and Cillessen, 2002; Lease et al., 2020). In addition, we predicted that children’s expectations about wealth and popularity groups would further depend on the group’s status level. Although evidence suggests that wealth and popularity are associated, children may be more likely expect a popular individual to have a large social network than a wealthy individual. Therefore, a less popular individual might socially profit from a popular peer to a greater extent than they would from a wealthy peer. While children expect there to be benefits from affiliating with wealthy (Ahl and Dunham, 2019; Ahl et al., 2019) and popular peers (Dijkstra et al., 2010; Cillessen and Marks, 2011; Lease et al., 2020), these expectations for wealthy children may be specific to material resources (Ahl and Dunham, 2019). For instance, they may be expected to share more than a poor individual due to having more resources to spare, rather than due to a broader prosocial tendency. In contrast, children expect popular individuals to help others in need and mediate conflict between others (Cillessen and Marks, 2011; Lease et al., 2020). For this reason, we included measures to examine children’s associations between wealth and popularity with prosocial helping and sharing behavior as an exploratory part of our investigation to examine children’s relative associations of wealth and popularity status groups with prosocial behavior.

**H4:** Lastly, we predicted that the effects of status and group norms would become increasingly pronounced with age. During middle childhood (ages 5–7 children generally have positive perceptions of high-status wealth and popularity groups (Cillessen and Marks, 2011; Shutts et al., 2016; Enright et al., 2020). However, by late childhood (ages 8–10) children attribute selfish motives to wealthy groups (Elenbaas and Killen, 2019) and overt and relational aggression to popular groups (Sandstrom and Cillessen, 2006). Previous research also shows between middle and late childhood, children’s understanding of how groups function (e.g., considerations of status, threat, group loyalty) becomes increasingly advanced (Nesdale et al., 2005; Abrams and Rutland, 2008; Abrams et al., 2009; Rutland et al., 2010). Evidence suggests that this is due, in part, to advanced perspective-taking abilities that emerge after the age of 8 (Banerjee, 2000) and allow children to better predict mental states within and between groups (Abrams et al., 2009). In addition, they become better at simultaneously weighing competing factors, such as the dynamics between status groups, norms, and their own personal preferences, when strategically reasoning about intergroup encounters (Abrams et al., 2003a; Killen and Rutland, 2011; Mulvey, 2016). The current study compared 5- to 7-year-old children’s expectations to those of 8- to 10-year-old children in order to examine differences in children’s conceptions of wealth and popularity status in relation to changes in their understanding of group dynamics and developing cognitive abilities.

## Materials and Methods

### Participants

The study included 165 5- to 10-year-old children (52.7% female, *M*_*age*_ = 7.72 years). Participants’ racial-ethnic background was indicated by parental report as follows: 60% White, 14.5% Black, 8.5% Latinx, 3.6% Asian, 6.1% multiethnic, 3.6% other, and 6% undisclosed. Participants were recruited from afterschool programs in the Mid-Atlantic region of the United States and through online venues. Identical protocol was used to test participants in-person and *via* Zoom, an online video conferencing software. All participants were shown colorful illustrations on a computer screen and interviewed individually by a researcher face-to-face.

### Design

The study utilized a 2 (Status Dimension: wealth, popularity) × 2 (Status Composition: low-status protagonist with high-status group, high-status protagonist with low-status group) × (Participant Age: 5–7, 8–10) × 2 (Gender: female, male) × 2 (Norm Presentation Order: inclusive first, exclusive first) × 2 (Norm: inclusive, exclusive) mixed design with repeated measures on the last factor. An *a priori* power analysis conducted in G*Power (Faul et al., 2009) determined that a sample size of 160 participants would be required to detect an effect size of *f* = 0.22 with 80% power, based on previous research utilizing similar designs which found effect sizes of *η_*p*_^2^* = 0.04 and 0.055 (Nesdale and Lawson, 2011; McGuire et al., 2015). This number was subsequently rounded up to include 165 participants in order to account for counterbalancing and potential exclusion from the final analyses due to reasons such as experimental error or attrition. In this study, all participants finished the protocol and there were no errors or attrition.

### Procedure

Participants were randomly assigned to one of four between-subjects conditions based on status composition (low-status protagonist/high-status group vs. high-status protagonist/low-status group) and status dimension (wealth, popularity). Participants were first introduced to a protagonist, who was described by their status dimension and level.

#### Wealth Status Descriptions

For participants in the wealth condition, status was depicted in terms of the target’s monetary resources, type of car, and type of house. Participants who saw a low-wealth target were told, “This is [protagonist/host]. [Protagonist/host]’s family has very of money. They drive a car like this, and they live in a house like this.” Low-wealth characters were shown with a small stack of dollar bills, an old rusty car, and a small and modest looking house. Participants who saw a high-wealth character and told, “This is [protagonist/host]. [Protagonist/host]’s family has lots and lots of money. They drive a car like this, and they live in a house like this.” High-wealth characters were shown with a large stack of dollar bills, a new luxury sports car, and a large and expensive looking house. The depictions were comparable to previous studies examining children’s conceptions of wealth (Mistry et al., 2015; Elenbaas and Killen, 2019; Burkholder et al., 2020).

#### Popularity Status Descriptions

For participants in the popularity condition, status was depicted in terms of friend group size (two = “low-popularity”; ten = “high-popularity), visibility, and influence. Participants who saw a low-popularity target were told, “This is [protagonist/host]. [Protagonist/host]has a friend group like this. Only a few kids know who [Protagonist/Host] is. At recess, [protagonist/host] always joins what someone else is doing.” Participants who saw a high-popularity target were told, “This is [Protagonist/Host]. [Protagonist/Host] has a friend group like this. All of the other kids know who [Protagonist/Host] is. At recess, a lot of kids always want to do what [Protagonist/Host]is doing.” The depictions were designed to be comparable to the wealth manipulation and were adapted from sociometric descriptions of popularity (Lease et al., 2002).

Participants were told that the protagonist was going to attend two birthday parties for two different peers (i.e., the hosts). The first party vignette was introduced by describing the host as being the opposite status level (same dimension) from the protagonist using the descriptions from above. Participants were informed that, apart from the protagonist, all of the other party attendees (i.e., the group) were the same status as the host (i.e., wealth: “Other kids with [very little/lots and lots] of money are going to the party”; popularity: “Other kids with [only a few/a lot of] friends are going to [Host]’s party”). The protagonist and host were both gender-matched to the participant to control for potential confounds with gender preferences.

#### Trait Associations

In order to examine children’s associations with wealth and popularity status participants in each of the four conditions made inferences about the host’s traits: wealth (“How wealthy is [Host]?”); popularity (“How many friends does [Host] have?”); sharing (“How often does [Host] help other kids who are sad and lonely?”); and helping (“How often does [Host] share the things he/she has with other kids?”) For each of these measures, participants indicated their responses on a 4-point Likert-type scale. The wealth measure served as a manipulation check in the two wealth conditions. Similarly, the popularity measure served as a manipulation check in the two popularity conditions.

#### Group 1 Norm Manipulation

Following the trait measures, participants the heard that the host held either an inclusive or exclusive norm regarding their status group.

For the inclusive host, participants heard, “[Host] says they like to be friends with kids who have any amount of [money/friends]. Some of their friends have only a [little bit of money/few friends] and some of their friends have a lot of [money/friends]. [Host] doesn’t think it matters how [much money/many friends] other kids have and they like kids who have any amount of [money/friends].

For the exclusive host, participants heard, “[Host] says they only like to be friends with kids who have [the same amount] of [money/friends]. None of their friends have [the opposite amount] of [money/friends] and all of their friends have [the same amount] of [money/friends]. [Host] thinks it really matters how much [money/friends] other kids have and they only like kids who have [the same amount] of [money/friends].”

#### Expectations for Social Resources

To examine how social status and normative information influences children’s expectations about acquiring social resources in cross-status encounters, participants predicted the group’s attitudes toward the protagonist (“How much will the other kids at this party like [Protagonist]?”), the protagonist’s enjoyment (“How much fun do you think the party will be for [Protagonist]?”), and group inclusion of the protagonist. For the attitude and enjoyment measures, participants indicated their responses on a 4-point Likert-type scale. For the inclusion measure, six targets (gender-matched to the participant) were displayed in an array and participants were told, “Here are some kids from the party. They’re each going to have their own birthday parties later this year.” Each target was then displayed individually and participants were asked, “Do you think this kid will invite [Protagonist] to their birthday party?” The number of “yes” responses (0–6) were recorded as a raw score.

Since we did not predict differences between these three measures, we created a composite score from participant ratings of group attitudes toward the protagonist, the protagonist’s enjoyment, and inclusion of the protagonist. For each measure, raw scores were transformed into *z*-scores and subsequently added to create a composite “expectations for acquiring social resources” score.

#### Group 2 Norm Manipulation

Next, the second party was introduced. Similar to the first vignette, the host and group were described as being the opposite status from the protagonist. However, participants were told that the second host held the opposite norm as the first host regarding their status group (host/group are same status in both vignettes). For this vignette, participants again predicted the group’s attitudes toward the protagonist, the protagonist’s enjoyment, and group inclusion of the protagonist. The order in which the participant received the inclusive or exclusive host in the first vignette was counterbalanced.

### Data Analytic Plan

Data were analyzed using the lme4 package for mixed-effects models in R (Bates et al., 2015; R Core Team, 2017). Preliminary analyses did not find significant effects of the interview method (i.e., in-person vs. online), gender, or the presentation order of the norm vignettes, which were unrelated to our hypotheses (*ps* > 0.05). Therefore, these variables were excluded from subsequent analyses. To test trait associations with wealth and popularity, we examined the effect of status dimension, status level, and participant age on ratings of the target’s wealth, popularity, sharing behavior, and helping behavior using analysis of variance (ANOVA).

The expectations for acquiring social resources composite score had acceptable internal consistency (3 items; α = 0.74). Thus, in order to test predictions about acquiring social resources, we examined the effect of status dimension, status level, group norm, and participant age on children’s expectations of social resources using mixed ANOVA with group norm as the within-subjects factor (see Supplementary Material for separate analyses by item). For each model, pairwise comparisons of the estimated marginal means were used to test expected differences between the factors and Bonferroni *post-hoc* tests were conducted to control for Type I errors.

## Results

### Associations Between Wealth and Popularity

**FIGURE 1:**
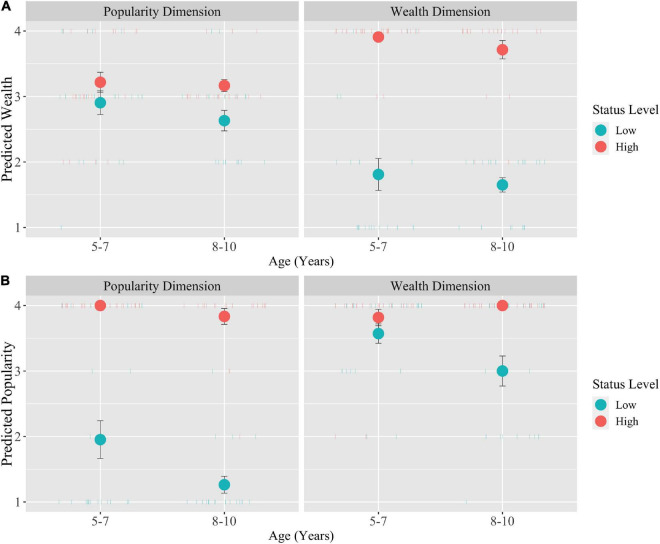
Children’s associations between wealth and popularity (with standard error bars). **(A)** Predicted wealth as a function of age, status dimension, and status level. **(B)** Predicted popularity as a function of age, status dimension, and status level.

First, we confirmed that the status descriptions use in the study effectively manipulated children’s beliefs about the targets’ wealth and popularity status. Children rated the high-wealth target (*M* = 3.81, *SE* = 0.08) as wealthier than the low-wealth target (*M* = 1.74, *SE* = 0.14), *t*(161) = 13.70, *p* < 0.001 (Figure 1A, Wealth Dimension). Children also rated the high-popularity target (*M* = 3.93, *SE* = 0.05) as more popular than the low-popularity target (*M* = 1.62, *SE* = 0.17), *t*(161) = 13.80, *p* < 0.001 (Figure 1B, Popularity Dimension).

As predicted (H1), we found a bidirectional association between wealth and popularity dimensions. An interaction between status dimension and status level on ratings of the target’s wealth, *F*(1, 157) = 58.22, *p* < 0.001, $\eta_{p}^{2}$ = 0.27 (Figure 1A, Popularity Dimension), revealed that children rated high-popularity targets (*M* = 3.20, *SE* = 0.09) as more wealthy than low-popularity targets (*M* = 2.78, *SE* = 0.12), *t*(161) = 2.72, *p* < 0.001. Similarly, there was an interaction between status dimension and status level on ratings of the target’s popularity, *F*(1,157) = 56.70, *p* < 0.001, $\eta_{p}^{2}$ = 0.27 (Figure 1B, Wealth Dimension), such that children rated high-wealth targets (*M* = 3.91, *SE* = 0.07) as more popular than low-wealth targets (*M* = 3.29, *SE* = 0.14), *t*(161) = 3.76, *p* < 0.001.

Participants’ wealth ratings did not significantly differ across age groups. However, there was an interaction between age and status level on popularity ratings, *F*(1,157) = 6.84, *p* < 0.01, $\eta_{p}^{2}$ = 0.04. Participants did not differ by age in how they rated high-status targets, but older children (*M* = 2.15, *SE* = 0.19) rated low-status targets as significantly less popular than did young children (*M* = 2.76, *SE* = 0.20), *t*(161) = 2.94, *p* < 0.01.

### Expectations About Acquiring Social Resources

**FIGURE 2:**
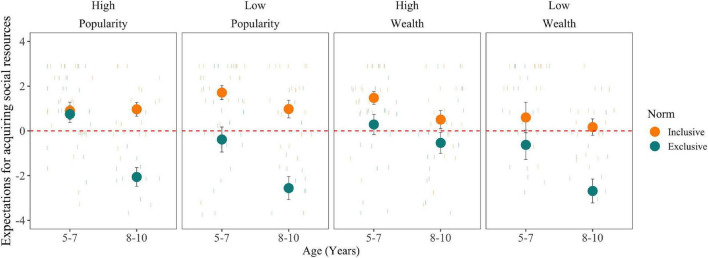
Children’s expectations for acquiring social resources as a function of norm, status dimension, status level, and participant age (with standard error bars). Expectations for acquiring social resources are based on a composite of *z*-scores for children’s predictions of the group’s attitudes toward the protagonist, the protagonist’s enjoyment, and the group’s inclusion of the protagonist. A score of zero indicates the mean of each sub-measure.

Children’s expectations about acquiring social resources are shown in Figure 2. Overall, children had greater expectations for the target to acquire social resources from a group that held norm of inclusion (*M* = 0.93, *SE* = 0.15) rather than from group that held and norm of exclusion (*M* = −0.93, *SE* = 0.20), *F*(1,157) = 91.36, *p* < 0.001, $\eta_{p}^{2}$ = 0.40. Although we expected this effect to be influenced by the group’s status level, we did not find support for this prediction (H2). Children’s expectations about a high-status group (*M* = 0.09, *SE* = 0.18) and a low-status group (*M* = −0.09, *SE* = 0.20) did not differ significantly. In addition, there were no significant interactive effects of status level on children’s expectations for acquiring social resources. Children’s expectations were slightly greater for a high-status inclusive group (*M* = 1.15, *SE* = 0.18) than for a low-status inclusive group (*M* = 0.69, *SE* = 0.24) but they did not differ from chance.

However, consistent with our predictions (H3), there was a significant main effect of status dimension, *F*(1,157) = 5.90, *p* = 0.02, $\eta_{p}^{2}$ = 0.04, and a interaction between norm and status dimension on children’s expectations for acquiring social resources, *F*(1,157) = 7.40, *p* < 0.001, $\eta_{p}^{2}$ = 0.05. Overall, children had lower expectations in the wealth dimension (*M* = −0.33, *SE* = 0.21) than in the popularity dimension (*M* = 0.34, *SE* = 0.16). When the group held a norm of inclusion, children exhibited similar expectations across both dimensions. However, the negative effects of a norm of exclusion on children’s expectations for acquiring resources were particularly pronounced for wealth groups (*M* = −1.54, *SE* = 0.30) compared to popularity groups (*M* = −0.30, *SE* = 0.24) independent of their status level.

Although we speculated that this finding might be due to differences in children’s associations of wealth and popularity with prosocial behavior, we did not find evidence for this. Participants generally viewed the target positively regardless of their status dimension or level. However, participant age did influence the extent to which children associated a target with sharing, *F*(1,157) = 15.68, *p* < 0.001, $\eta_{p}^{2}$ = 0.09, and helping, *F*(1,157) = 14.30, *p* < 0.001, $\eta_{p}^{2}$ = 0.08. Younger children (*M* = 3.29, *SE* = 0.10) were more likely to expect targets to share material resources than older children *M* = 2.77, *SE* = 0.08) and younger children were also more likely to expect targets to help others in need (*M* = 3.37, *SE* = 0.09), than older children (*M* = 2.88, *SE* = 0.09).

We found partial evidence for our hypothesis that the effect of norms and status become more pronounced with age. Overall, older children (*M* = −0.67, *SE* = 0.19) had lower expectations for a target to acquire social resources than younger children (*M* = 0.60, *SE* = 0.18), *F*(1,157) = 22.54, *p* < 0.001, $\eta_{p}^{2}$ = 0.13. There was also an interaction of participant age and norm on expectations for acquiring social resources, *F*(1,157) = 13.41, *p* < 0.001, $\eta_{p}^{2}$ = 0.08. When the group held an inclusive norm, older children (*M* = 0.09, *SE* = 0.18) and younger children did not differ in their expectations, *p* > 0.05. However, when the group held an exclusive norm, older children (*M* = −1.93, *SE* = 0.26), expected fewer resources than younger children (*M* = 0.02, *SE* = 0.26), *t*(157) = 6.00, *p* < 0.001. Neither status dimension nor status level, however, interacted with participant age.

## Discussion

Previous research suggests that children infer rank across multiple dimensions of social status and favor high-status groups over low-status groups. We speculated that children’s biases could be, in part, due to associations between wealth and popularity dimensions and expectations about the benefits of intergroup affiliation might contribute to children’s biases. The present study extended previous research by comparing the relative strength of children’s associations between wealth and popularity status, and examining children’s expectations acquiring social resources (i.e., positive attitudes, enjoyment, and inclusion) through cross-status affiliation in wealth and popularity contexts. Two primary novel findings emerged. First, we found that children positively associated wealth and popularity status. Children viewed high-popularity targets as wealthier than low-popularity targets (provided with no information about wealth) and viewed high-wealth targets as more popular than low-wealth targets (provided with no information about popularity). This finding is consistent with previous work showing that children associate features of wealth and popularity (Lease et al., 2002; Charafeddine et al., 2015; Shutts et al., 2016; Gülgöz and Gelman, 2017; Enright et al., 2020). However, we extend previous research by providing evidence of a bidirectional association and comparing the relative strength of inferences across these two dimensions.

Children inferred popularity from wealth descriptions more strongly than they inferred wealth from popularity descriptions. They viewed high-wealth targets as equally popular as high-popularity targets but did not view high-popularity targets as equally wealthy as high-wealth targets. Moreover, older children distinguished between high- and low-wealth targets in their inferences about popularity to a greater extent than younger children. Evidence suggests that young children make inferences on the basis of one’s quantity of physical resources such as possessions and friends (Pun et al., 2016; Ahl and Dunham, 2019). However, they may view non-physical resources as less indicative of status. For example, 3- to 4-year-old children view individuals who control access to material resources as powerful, but do not view an individuals who gives orders as powerful until 7–9 years of age (Gülgöz and Gelman, 2017). In addition, children in grades 3–5 view peers who influence others’ behavior and set social norms as high-status (Lease et al., 2020). We suspect that young children do not necessarily view social visibility and influence over others’ behavior as attributes that contribute to status while older children likely do. However, we can only speculate about children’s relative prioritization of physical and non-physical resources. More investigation is needed to determine whether children distinguish between these types of resources.

The second novel finding was that norms of exclusion diminished children’s expectations for acquiring social resources from wealth and popularity groups but was more pronounced in wealth contexts. Surprisingly, we did not find evidence that children’s expectations were dependent on the group’s status level. This is in contrast to an overwhelming body of research that suggests that considerations of wealth status (Woods et al., 2005; Sigelman, 2012; Li et al., 2014; Mistry et al., 2015; Shutts et al., 2016; Ahl and Dunham, 2019; Ahl et al., 2019; Enright et al., 2020) and popularity status (LaFontana and Cillessen, 2002; Lease et al., 2002, 2020; Cillessen and Marks, 2011; Sandstrom, 2011) do indeed impact children’s attitudes an expectations about others. Our results do not imply that children’s broader evaluations, or even their more specific expectations about acquiring social resources, are not informed by status differences. In fact, additional analyses conducted on each independent social resources sub-measure found that children expected that attending a low-wealth party would be significantly less enjoyable than attending a party with a high-wealth or either type of popularity group (see Supplementary Material). Rather, our findings suggest that group norms and status dimension are relatively more informative for children’s expectations about acquiring resources than status level. Norms of inclusion and exclusion had a particularly powerful effect on children’s expectations overall, but operated differently for wealth and popularity.

We suspect that children more readily generalized the host’s exclusive preferences to other wealth group members than they did to popularity group members due to their pre-existing beliefs about wealth groups. Regardless of whether children make more favorable assumptions about high- or low-wealth groups, they may generally believe that both groups prefer their in-group. This explanation would be consistent with evidence that children expect peers to prefer affiliation with their own wealth group even those wealth in-group members are out-group members on another dimension such as race (Burkholder et al., 2021). Also in line with evidence that norms of inclusion can mitigate prejudice (Nesdale et al., 2007; Nesdale and Lawson, 2011), our findings suggest that although children may hold pre-existing beliefs about wealth groups are exclusive, norms of inclusion may broadly reduce their perceptions of social barriers between high- and low-status groups.

However, given that the current study already included multiple factors that could influence children’s, we could not control for the influence of norms, for instance, by including a condition that would allow us to examine children’s expectations in a more neutral context (i.e., without the influence of an explicit norm). Therefore, we could not draw conclusion about the relative impact of norms on children’s pre-existing expectations about cross-status affiliation. Children may hold different stereotypes about how inclusive or exclusive wealth and popularity groups are in general. For instance, in the absence of explicit information, children could expect wealth peers to be exclusive while viewing popular peers as inclusive. If this were the case, then our finding that children’s expectations about an inclusive wealth group were just as optimistic as they were for an inclusive popularity group would suggest that the norm was relatively more powerful for wealth groups than for popularity groups.

This limitation of the study design may have also obscured potential status level differences. The negative effects of an exclusive norm may could have been due to negative assumptions about the group’s status or the protagonist’s status. Children differentiate more between malevolent and benevolent forms of status (Gülgöz and Gelman, 2017; Kajanus et al., 2020). Although they infer similar rank between prestigious and dominant targets, children expect a character to prefer affiliation with a prestigious target who shares their opinion when asked over a dominant target who forces their opinion (Kajanus et al., 2020). Yet, the participants in our sample generally rated all targets positively prior to hearing the norm manipulation so we do not believe that the main effects of the norm were strongly based on children’s assumptions that a target would be more or less likely to acquire social resources from a certain status group. However, more evidence is needed to understand why children’s expectations were lower for an exclusive wealth group than an exclusive popularity group and future research should investigate how children’s expectations about similarly ranked wealth and popularity groups might differ in more ambiguous contexts.

In addition to the previously described findings, we found age-related differences in children’s expectations for acquiring social resources. The participant age groups included in this study held similar expectations for inclusive wealth and popularity groups, but 8- to 10-year-old children’s expectations for acquiring social resources were significantly lower than 5- to 7-year-old children’s expectation. This is consistent with previous evidence that children become increasingly sensitive to group norms with age (Nesdale et al., 2005; Abrams and Rutland, 2008; Abrams et al., 2009; Rutland et al., 2010; Rutland and Killen, 2015). However, we did not find evidence that age differences in children’s expectations about obtaining social resources were specifically linked status groups based on wealth and popularity. This in contrast to evidence that children’s conceptions of wealth and popularity status change between middle- and late-childhood (Cillessen and Marks, 2011; Shutts et al., 2016; Enright et al., 2020). It’s possible that the interaction of norms and participant age could be explained by a stronger positivity bias among younger children than among older children, however, there are many instances in which younger children are seemingly more pessimistic or negative than older children in in their trait attributions and expectations for behavior (Aboud, 2008). For example, younger children are more willing than older children and adults to condone retribution and punish a transgressor regardless of intention (Mulvey et al., 2020).

In the current study, children’s expectations about wealth and popularity dimensions appear to be similarly informed by norms and their prioritization of norms increases with age. However, we suspect that the effects of how younger and older children differentially consider norms in relation to different aspects of status may be too subtle to detect between middle and late childhood (Nesdale et al., 2007; McGuire et al., 2019). Adolescents’ (13–16 years), but not children’s (7–11 years), resource allocations to disadvantaged in-groups than disadvantaged out-groups (i.e., low-status) were dependent on group norms (McGuire et al., 2019). In other words, participants had to coordinate considerations of status level, how each norm applied to each level, and how their own group membership interacted with these factors. Similarly, the current study asked children to consider these same factors in relation to different status dimensions instead of group memberships. The added consideration of the group membership distinction in the McGuire et al. (2019) study and of the dimension distinction in the current study, in conjunction with group norms, may be beyond children’s abilities to systematically coordinate in late childhood. Given that with age, children differentially coordinate how they apply norms to different groups (including those based on status level), we posit that the absence of an interactive effect on either status level or status dimension had more to do with a limited ability to coordinate multiple competing factors, rather than due to a limitation in children’s ability to differentiate between dimensions and levels of status.

## Conclusion

Reasoning about status can become rather complex, perhaps overwhelmingly so for children, given its multifaceted features. Therefore, children’s expectations about status appear to be highly dependent social contexts. It’s possible that in some contexts (i.e., regarding material resources) children’s expectations about acquiring resources may be more informed by the relative status rank between groups than the dimension of status. In the context of the current study, exclusive norms across status dimensions appeared to lead to lower expectations for acquiring social resources than exclusive norms across groups of different status levels. This is a promising finding because sheds light on the possibility for mitigating children’s biases toward high-status groups. Emphasizing positive qualities among low-status groups or negative qualities among high-status groups across broader dimensions may, to some extent, reduce children’s tendency to favor high-status groups more generally. Understanding the nuances in how children prioritize multiple features of status is thus, critical to devising methods that mitigate status biases.

## Data Availability Statement

The datasets presented in this article are not readily available because they are part of an ongoing research project. Requests to access the datasets should be directed to KY.

## Ethics Statement

The studies involving human participants were reviewed and approved by the University of Maryland’s Institutional Review Board (1470874-6). Written informed consent to participate in this study was provided by the participants’ parent/legal guardian.

## Author Contributions

KY and JG contributed to data collection. KY conducted the statistical analyses and wrote the first draft of the manuscript. All authors contributed to the design of study and revisions, read, and approved the submitted version.

## Conflict of Interest

The authors declare that the research was conducted in the absence of any commercial or financial relationships that could be construed as a potential conflict of interest.

## Publisher’s Note

All claims expressed in this article are solely those of the authors and do not necessarily represent those of their affiliated organizations, or those of the publisher, the editors and the reviewers. Any product that may be evaluated in this article, or claim that may be made by its manufacturer, is not guaranteed or endorsed by the publisher.

## References

[B1] Aboud F. E. (2008). “A social-cognitive developmental theory of prejudice,” in *Handbook of Race, Racism, and the Developing Child*, eds Quintana S. M. McKown C. (New Jersey, NJ: John Wiley & Sons).

[B2] Abrams D. Rutland A. (2008). “The development of subjective group dynamics,” in *Intergroup Attitudes and Relations in Childhood and Throughout Adulthood*, eds Levy S. R. Killen M. (Oxford: Oxford University Press). 47–65.

[B3] Abrams D. Rutland A. Cameron L. (2003a). The development of subjective group dynamics: children’s judgments of normative and deviant in-group and out-group individuals. *Child Dev.* 74 1840–1856. 10.1046/j.1467-8624.2003.00641.x 14669899

[B4] Abrams D. Rutland A. Cameron L. Marques J. (2003b). The development of subjective group dynamics: when in-group bias gets specific. *Br. J. Dev. Psychol.* 21 155–176. 10.1348/026151003765264020

[B5] Abrams D. Rutland A. Pelletier J. Ferrell J. M. (2009). Children’s group nous: understanding and applying peer exclusion within and between groups. *Child Dev.* 80 224–243. 10.1111/j.1467-8624.2008.01256.x 19236403

[B6] Ahl R. E. Dunham Y. (2019). “Wealth makes many friends”: children expect more giving from resource-rich than resource-poor individuals. *Child Dev.* 90 524–543. 10.1111/cdev.12922 28832977

[B7] Ahl R. E. Duong S. Dunham Y. (2019). Children employ wealth cues when predicting others’ sharing. *Dev. Psychol.* 55 303–314. 10.1037/dev0000643 30525834

[B8] Anderson C. Hildreth J. A. D. Howland L. (2015). Is the desire for status a fundamental human motive? A review of the empirical literature. *Psychol. Bull.* 141 574–601. 10.1037/a0038781 25774679

[B9] Banerjee R. (2000). The development of an understanding of modesty. *Br. J. Dev. Psychol.* 18 499–517. 10.1348/026151000165823

[B10] Baron A. Banaji M. (2009). Evidence of system justification in young children. *Soc. Pers. Psychol. Compass* 3 918–926. 10.1111/j.1751-9004.2009.00214.x

[B11] Bates D. Mächler M. Bolker B. Walker S. (2015). Fitting linear mixed-effects models using lme4. *J. Stat. Softw.* 67:1048. 10.18637/jss.v067.i01

[B12] Bennett M. (2014). Intergroup social exclusion in childhood: forms, norms, context, and social identity. *J. Soc. Issues* 70 183–195. 10.1111/josi.12054

[B13] Brey E. Shutts K. (2015). Children use nonverbal cues to make inferences about social power. *Child Dev.* 86 276–286. 10.1111/cdev.12334 25521913 PMC4795924

[B14] Burkholder A. R. Elenbaas L. Killen M. (2020). Children’s and adolescents’ evaluations of intergroup exclusion in interracial and interwealth peer contexts. *Child Dev.* 91 e512–e527. 10.1111/cdev.13249 31144306 PMC9048094

[B15] Burkholder A. R. Elenbaas L. Killen M. (2021). Giving priority to race or wealth in peer group contexts involving social inclusion. *Dev. Psychol.* 57 651–661. 10.1037/dev0001178 34166012 PMC8238375

[B16] Cain K. M. Heyman G. D. Walker M. E. (1997). Preschoolers’ ability to make dispositional predictions within and across domains. *Soc. Dev.* 6 52–75. h0035080 10.1111/J.1467-9507.1997.TB00094.X

[B17] Chafel J. A. Neitzel C. (2005). Young children’s ideas about the nature, causes, justification, and alleviation of poverty. *Early Child. Res. Q.* 20 433–450. 10.1016/j.ecresq.2005.10.004

[B18] Charafeddine R. Mercier H. Clément F. Kaufmann L. Berchtold A. Reboul A. (2015). How preschoolers use cues of dominance to make sense of their social environment. *J. Cogn. Dev.* 16 587–607. 10.1080/15248372.2014.926269

[B19] Cillessen A. H. N. (2009). “Sociometric methods,” in *Handbook of Peer Interactions, Relationships, and Groups*, eds Rubin K. H. Bukowski W. M. Laursen B. (New York, NY: The Guilford Press). 82–99.

[B20] Cillessen A. H. N. Marks P. E. L. (2011). “Conceptualizing and measuring popularity,” in *Popularity in the Peer System*, eds Cillessen A. H. N. Schwartz D. Mayeux L. (New York, NY: The Guilford Press). 25–56.

[B21] Dijkstra J. K. Cillessen A. H. N. Lindenberg S. Veenstra R. (2010). Basking in reflected glory and its limits: why adolescents hang out with popular peers. *J. Res. Adolesc.* 20 942–958. 10.1111/j.1532-7795.2010.00671.x

[B22] Dunham Y. Baron A. S. Carey S. (2011). Consequences of “minimal” group affiliations in children. *Child Dev.* 82, 793–811. 10.1111/j.1467-8624.2011.01577.x 21413937 PMC3513287

[B23] Dys S. P. Peplak J. Colasante T. Malti T. (2019). Children’s sympathy and sensitivity to excluding economically disadvantaged peers. *Dev. Psychol.* 55 482–487. 10.1037/dev0000549 30802100

[B24] Elenbaas L. Killen M. (2019). Children’s perceptions of economic groups in a context of limited access to opportunities. *Child Dev.* 90 1632–1649. 10.1111/cdev.13024 29333602 PMC11161858

[B25] Enright E. A. Alonso D. J. Lee B. M. Olson K. R. (2020). Children’s understanding and use of four dimensions of social status. *J. Cogn. Dev.* 21 573–602. 10.1080/15248372.2020.1797745

[B26] Faul F. Erdfelder E. Buchner A. Lang A.-G. (2009). Statistical power analyses using G*Power 3.1: tests for correlation and regression analyses. *Behav. Res. Methods* 41 1149–1160. 10.3758/BRM.41.4.114 19897823

[B27] Feldman D. C. (1984). The development and enforcement of group norms. *Acad. Manage. Rev.* 9 47–53. 10.5465/amr.1984.4277934

[B28] Gülgöz S. Gelman S. A. (2017). Who’s the boss? Concepts of social power across development. *Child Dev.* 88 946–963. 10.1111/cdev.12643 27739071

[B29] Hitti A. Killen M. (2015). Expectations about ethnic peer group inclusivity: the role of shared interests, group norms, and stereotypes. *Child Dev.* 86 1522–1537. 10.1111/cdev.12393 26154412

[B30] Hitti A. Mulvey K. L. (2021). The consequences of outgroup helping: children’s and adolescents’ reasoning. *J. Exp. Child Psychol.* 203 105013. 10.1016/j.jecp.2020.105013 33221661

[B31] Horn S. S. (2006). Group status, group bias, and adolescents’ reasoning about the treatment of others in school contexts. *Int. J. Behav. Dev.* 30 208–218. 10.1177/0165025406066721

[B32] Horwitz S. R. Shutts K. Olson K. R. (2014). Social class differences produce social group preferences. *Dev. Sci.* 17 991–1002. 10.1111/desc.12181 24702971 PMC4188807

[B33] Kajanus A. Afshordi N. Warneken F. (2020). Children’s understanding of dominance and prestige in China and the UK. *Evol. Hum. Behav.* 41 23–34. 10.1016/j.evolhumbehav.2019.08.002

[B34] Killen M. Rutland A. (2011). *Children and Social Exclusion: Morality, Prejudice, and Group Identity.* New York, NY: Wiley-Blackwell. 10.1002/9781444396317

[B35] LaFontana K. M. Cillessen A. H. N. (2002). Children’s perceptions of popular and unpopular peers: a multimethod assessment. *Dev. Psychol.* 38 635–647. 10.1037/0012-1649.38.5.635 12220043

[B36] LaFontana K. M. Cillessen A. H. N. (2010). Developmental changes in the priority of perceived status in childhood and adolescence. *Soc. Dev.* 19 130–147. 10.1111/j.1467-9507.2008.00522.x

[B37] Lansu T. A. M. Cillessen A. H. N. Karremans J. C. (2012). Implicit associations with popularity in early adolescence: an approach–avoidance analysis. *Dev. Psychol.* 48 65–75. 10.1037/a0025681 21942665

[B38] Leahy R. L. (1990). The development of concepts of economic and social inequality. *New Dir. Child Adolesc. Dev.* 1990 107–120. 10.1002/cd.23219904608 2348933

[B39] Lease A. M. Kennedy C. A. Axelrod J. L. (2002). Children’s social constructions of popularity. *Soc. Dev.* 11 87–109. 10.1111/1467-9507.00188

[B40] Lease A. M. Kwon K. Lovelace M. Huang H. (2020). Peer influence in elementary school: the importance of assessing the likeability of popular children. *J. Genet. Psychol.* 181 95–110. 10.1080/00221325.2020.1730744 32090707

[B41] Li V. Spitzer B. Olson K. R. (2014). Preschoolers reduce inequality while favoring individuals with more. *Child Dev.* 85 1123–1133. 10.1111/cdev.12198 24359582

[B42] McGuire L. Elenbaas L. Killen M. Rutland A. (2019). The role of in-group norms and group status in children’s and adolescents’ decisions to rectify resource inequalities. *Br. J. Dev. Psychol.* 37 309–322. 10.1111/bjdp.12274 30548276

[B43] McGuire L. Manstead A. S. R. Rutland A. (2017). Group norms, intergroup resource allocation, and social reasoning among children and adolescents. *Dev. Psychol.* 53 2333–2339. 10.1037/dev0000392 28933878

[B44] McGuire L. Rutland A. Nesdale D. (2015). Peer group norms and accountability moderate the effect of school norms on children’s intergroup attitudes. *Child Dev.* 86 1290–1297. 10.1111/cdev.12388 26082195

[B45] Mistry R. S. Brown C. S. White E. S. Chow K. A. Gillen-O’Neel C. (2015). Elementary school children’s reasoning about social class: a mixed-methods study. *Child Dev.* 86 1653–1671. 10.1111/cdev.12407 26300338

[B46] Mulvey K. L. (2016). Children’s reasoning about social exclusion: balancing many factors. *Child Dev. Perspect.* 10 22–27. 10.1111/cdep.12157

[B47] Mulvey K. L. Gönültaş S. Richardson C. B. (2020). Who Is to blame? Children’s and adults’ moral judgments regarding victim and transgressor negligence. *Cogn. Sci.* 44:e12833. 10.1111/cogs.12833 32274859

[B48] Nesdale D. (2008). “Peer group rejection and children’s intergroup prejudice,” in *Intergroup Attitudes and Relations in Childhood Through Adulthood*, eds Levy S. R. Killen M. (Oxford: Oxford University Press). 32–46.

[B49] Nesdale D. Dalton D. (2010). Children’s social groups and intergroup prejudice: assessing the influence and inhibition of social group norms. *Br. J. Dev. Psychol.* 29 895–909. 10.1111/j.2044-835X.2010.02017.x 21995743

[B50] Nesdale D. Lawson M. J. (2011). Social groups and children’s intergroup attitudes: can school norms moderate the effects of social group norms? *Child Dev.* 82 1594–1606. 10.1111/j.1467-8624.2011.01637.x 21883158

[B51] Nesdale D. Durkin K. Maass A. Kiesner J. Griffiths J. A. (2008). Effects of group norms on children’s intentions to bully. *Soc. Dev.* 17 889–907. 10.1111/j.1467-9507.2008.00475.x

[B52] Nesdale D. Maass A. Durkin K. Griffiths J. (2005). Group norms, threat, and children’s racial prejudice. *Child Dev.* 76 652–663. 10.1111/j.1467-8624.2005.00869.x 15892784

[B53] Nesdale D. Maass A. Kiesner J. Durkin K. Griffiths J. Ekberg A. (2007). Effects of peer group rejection, group membership, and group norms, on children’s outgroup prejudice. *Int. J. Behav. Dev.* 31 526–535. 10.1177/0165025407081479

[B54] Newheiser A.-K. Dunham Y. Merrill A. Hoosain L. Olson K. R. (2014). Preference for high status predicts implicit outgroup bias among children from low-status groups. *Dev. Psychol.* 50 1081–1090. 10.1037/a0035054 24219317 PMC3981896

[B55] Olson K. R. Shutts K. Kinzler K. D. Weisman K. G. (2012). Children associate racial groups with wealth: evidence from South Africa. *Child Dev.* 83 1884–1899. 10.1111/j.1467-8624.2012.01819.x 22860510 PMC3492517

[B56] Poorthuis A. M. G. Thomaes S. Denissen J. J. A. van Aken M. A. G. de Castro B. (2012). Prosocial tendencies predict friendship quality, but not for popular children. *J. Exp. Child Psychol.* 112 378–388. 10.1016/j.jecp.2012.04.002 22578912

[B57] Pun A. Birch S. A. J. Baron A. S. (2016). Infants use relative numerical group size to infer social dominance. *Proc. Natl. Acad. Sci.U.S.A* 113 2376–2381. 10.1073/pnas.1514879113 26884199 PMC4780600

[B58] R Core Team. (2017). *R: A Language and Environment for Statistical Computing.* Available online at: https://www.R-project.org. (accessed October 31, 2017)

[B59] Renno M. P. Shutts K. (2015). Children’s social category-based giving and its correlates: expectations and preferences. *Dev. Psychol.* 51, 533–543. 10.1037/a0038819 25706588

[B60] Rutland A. Killen M. (2015). A developmental science approach to reducing prejudice and social exclusion: intergroup processes, social-cognitive development, and moral reasoning. *Soc. Issues Policy Rev.* 9 121–154. 10.1111/sipr.12012

[B61] Rutland A. Cameron L. Milne A. McGeorge P. (2005). Social norms and self-presentation: children’s implicit and explicit intergroup attitudes. *Child Dev.* 76 451–466. 10.1111/j.1467-8624.2005.00856.x 15784093

[B62] Rutland A. Killen M. Abrams D. (2010). A new social-cognitive developmental perspective on prejudice: the interplay between morality and group identity. *Perspect. Psychol. Sci.* 5 279–291. 10.1177/1745691610369468 26162160

[B63] Sandstrom M. J. (2011). “The power of popularity: influence processes in childhood and adolescence,” in *Popularity in the Peer System*, eds Cillessen A. H. N. Schwartz D. Mayeux L. (New York, NY: The Guilford Press). 219–244.

[B64] Sandstrom M. J. Cillessen A. H. N. (2006). Likeable versus popular: distinct implications for adolescent adjustment. *Int. J. Behav. Dev.* 30 305–314. 10.1177/0165025406072789

[B65] Shutts K. Brey E. L. Dornbusch L. A. Slywotzky N. Olson K. R. (2016). Children use wealth cues to evaluate others. *PLoS One* 11:e0149360. 10.1371/journal.pone.0149360 26933887 PMC4774995

[B66] Sigelman C. K. (2012). Rich man, poor man: developmental differences in attributions and perceptions. *J. Exp. Child Psychol.* 113 415–429. 10.1016/j.jecp.2012.06.011 22858091

[B67] Woods T. A. Kurtz-Costes B. Rowley S. J. (2005). The development of stereotypes about the rich and poor: age, race, and family income differences in beliefs. *J. Youth Adolesc.* 34 437–445. 10.1007/s10964-005-7261-0

[B68] Zhang X. Corbit J. Xiao X. Xu L. Wei B. Li Y. (2021). Material and relational asymmetry: the role of receivers’ wealth and power status in children’s resource allocation. *J. Exp. Child Psychol.* 208:105147. 10.1016/j.jecp.2021.105147 33862531

