# Supplementary material for: Group Norms Influence Children’s Expectations About Status Based on Wealth and Popularity
Source: Front Psychol. 2022 May 11;13:816205. doi: 10.3389/fpsyg.2022.816205 (PMC9131005; doi:10.3389/fpsyg.2022.816205)
Supplement: Supplementary file 1 [file Data_Sheet_1.PDF]

Supplementary Material

1 Summary

"*Group norms influence children's expectations about status based on wealth and popularity*" investigated children's expectations about acquiring social resources through cross-status affiliation in wealth and popularity contexts. The expectations for acquiring social resources measure was calculated from an additive composite score of 3 sub-measures' z-scores. The composite score had acceptable internal consistency (3 items; α = .74) and was used for the analyses reported in the main manuscript. Here, we report separate analyses for the individual sub-measures: 1) expectations for positive group attitudes towards the protagonist, 2) expectations for the protagonist's enjoyment, and 3) expectations for group inclusion of the protagonist. For a full description of the sub-measures, please refer to the main manuscript.

2 Data Analytic Plan

Data were analyzed using the lme4 package for mixed-effects models in R (Bates et al., 2015; R Core Team, 2017). We examined the effect of status dimension, status level, group norm, and participant age on each sub-measure using analysis of variance with group norm as the within-subjects factor. For each model, pairwise comparisons of the estimated marginal means were used to test expected differences between the factors and Bonferroni post-hoc tests were conducted to control for Type I errors.

3 Expectations for acquiring social resources sub-measures

3.1 Expectations for positive group attitudes towards the protagonist
(Supplementary Figure 1)

We found main effects of norm, $F(1, 157) = 83.69$, $p < .001$, $\eta_p^2 = .35$, status dimension, $F(1, 157) = 5.98$, $p < .05$, $\eta_p^2 = .04$, and participant age, $F(1, 157) = 18.36$, $p < .001$, $\eta_p^2 = .10$ on children's expectations for positive group attitudes towards the protagonist. Overall, children exhibited higherr expectations when the group held a norm of inclusion ($M = 3.34$, $SE = .06$) than when the group held a norm of exclusion ($M = 2.51$, $SE = .08$), in the popularity dimension ($M = 3.06$, $SE = .07$) than in the wealth dimension ($M = 2.80$, $SE = .08$), and among 5- to 7-year-old children ($M = 3.13$, $SE = .08$) than among 8- to 10-year-old children ($M = 2.69$, $SE = .08$). There were no significant main or interactive effects of status level.

There was a significant interaction of norm and status dimension on children's expectations for positive group attitudes towards the protagonist, $F(1, 157) = 5.28$, $p < .05$, $\eta_p^2 = .03$. When the group held a norm of inclusion, children's expectations did not differ between the wealth dimension ($M = 3.32$, $SE = .09$) and popularity dimension ($M = 3.36$, $SE = .07$), $t(326) = 0.26$, $p > .05$. However, when the group held a norm of exclusion, children had lower expectations for positive attitudes in the wealth dimension ($M = 2.27$, $SE = .12$) than the popularity dimension ($M = 2.75$, $SE = .11$), $t(326) = 3.43$, $p < .001$.

There was a significant interaction of norm and participant age on children's expectations for positive group attitudes towards the protagonist, $F(1, 157) = 4.95, p < .05, \eta_p^2 = .03$. When the group held a norm of inclusion, 5- to 7-year-old children's expectations ($M = 3.45, SE = .08$) did not differ from 8- to 10-year-olds ($M = 3.22, SE = .07$), $t(326) = 1.68, p > .05$. However, when the group held a norm of exclusion, 8- to 10-year-old children ($M = 2.17, SE = .10$) had lower expectations for positive attitudes 5- to 7-year-old children ($M = 2.82, SE = .12$), $t(326) = 4.73, p < .001$.

3.2 Expectations for protagonist's enjoyment
(Supplementary Figure 2)

We found main effects of norm, $F(1, 157) = 61.54, p < .001, \eta_p^2 = .28$, status dimension, $F(1, 157) = 6.14, p < .05, \eta_p^2 = .04$, and participant age, $F(1, 157) = 21.27, p < .001, \eta_p^2 = .12$ on children's expectations for the protagonist's enjoyment. Overall, children exhibited greater expectations when the group held a norm of inclusion ($M = 3.45, SE = .07$) than when the group held a norm of exclusion ($M = 3.00, SE = .08$), in the popularity dimension ($M = 3.28, SE = .07$) than in the wealth dimension ($M = 3.00, SE = .08$), and among 5- to 7-year-old children ($M = 3.39, SE = .07$) than among 8- to 10-year-old children ($M = 3.87, SE = .08$). There was no significant main effect of status level.

There was a significant interaction of status dimension and status level on children's expectations for the protagonist's enjoyment at the party, $F(1, 157) = 7.52, p < .01, \eta_p^2 = .05$. Children's expectations did not differ between a high-popularity ($M = 3.23, SE = .11$) and low-popularity party ($M = 3.34, SE = .09$), $t(326) = -0.68, p > .05$, but children had lower expectations for a low-wealth ($M = 2.76, SE = .13$) than a high-wealth party ($M = 3.23, SE = .11$), $t(326) = 3.11, p < .01$. Moreover, children's expectations did not differ between a high-wealth ($M = 3.23, SE = .11$) and a high-popularity party ($M = 3.23, SE = .11$), $t(326) = -0.01, p > .05$, but children had lower expectations for the protagonist's enjoyment at a low-wealth ($M = 2.76, SE = .13$) than a low-popularity party ($M = 3.34, SE = .09$), $t(326) = 3.73, p < .001$.

There was a significant interaction of norm and participant age on children's expectations for the protagonist's enjoyment, $F(1, 157) = 16.14, p < .001, \eta_p^2 = .09$. When the group held a norm of inclusion, 5- to 7-year-old children's expectations ($M = 3.54, SE = .09$) did not differ from 8- to 10-year-olds ($M = 3.36, SE = .09$), $t(326) = 1.27, p > .05$. However, when the group held a norm of exclusion, 8- to 10-year-old children ($M = 2.37, SE = .12$) had lower expectations for the protagonist's enjoyment than 5- to 7-year-old children ($M = 3.23, SE = .10$), $t(326) = 6.01, p < .001$.

3.3 Expectations for group inclusion of the protagonist
(Supplementary Figure 3)

We found main effects of norm, $F(1, 157) = 20.61, p < .001, \eta_p^2 = .28$, and participant age, $F(1, 157) = 6.3q, p = .01, \eta_p^2 = .12$, on children's expectations for group inclusion of the protagonist. Overall, children exhibited greater expectations when the group held a norm of inclusion ($M = 4.55, SE = .13$) than when the group held a norm of exclusion ($M = 3.79, SE = .16$) and among 5- to 7-year-old children ($M = 4.44, SE = .14$) than among 8- to 10-year-old children ($M = 3.87, SE = .15$). There were no significant main effects of status dimension or status level.

There was a small, but significant effect of norm and status dimension on children's expectations for group inclusion, $F(1, 157) = 7.04$, $p < .01$, $\eta_p^2 = .01$. Children's expectations did not differ between and inclusive wealth group ($M = 4.65$, $SE = .18$) and an inclusive popularity group ($M = 4.43$, $SE = .18$), $t(326) = -0.79$, $p > .05$. However, children had lower expectations for an exclusive wealth group ($M = 3.45$, $SE = .24$) than an exclusive popularity group ($M = 4.15$, $SE = .19$), $t(326) = 2.46$, $p = .01$. In addition, children's expectations did not differ between an inclusive popularity ($M = 4.43$, $SE = .18$) and exclusive popularity group ($M = 4.15$, $SE = .19$), $t(326) = 0.99$, $p > .05$, but children had lower expectations for an exclusive wealth group ($M = 3.45$, $SE = .24$) than an inclusive wealth group ($M = 4.65$, $SE = .18$)), $t(326) = 4.29$, $p < .001$.

There was a significant effect of norm and participant age on children's expectations for group inclusion, $F(1, 157) = 4.49$, $p < .05$, $\eta_p^2 = .09$. Expectations about inclusive groups did not differ between 5- to 7-year-old children ($M = 4.64$, $SE = .19$) and 8- to 10-year-old children ($M = 4.44$, $SE = .18$), $t(326) = 1.47$, $p > .05$, but 8- to 10-year-old children had lower expectations ($M = 3.29$, $SE = .23$) than 5- to 7-year-old children ($M = 4.24$, $SE = .20$), $t(326) = 3.95$, $p < .001$.

4 Supplementary Figures

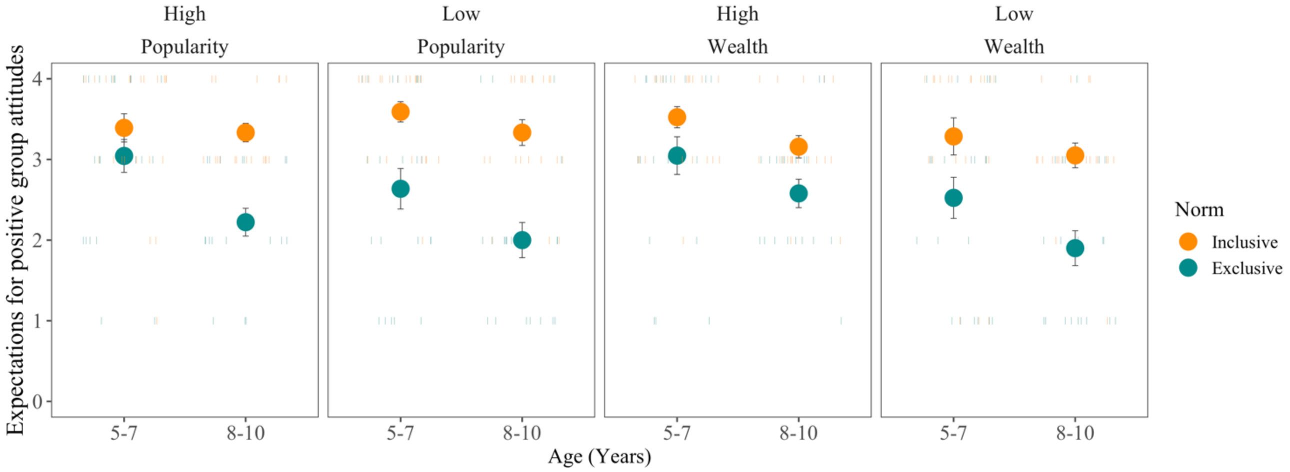

Supplementary Figure 1. Expectations for positive group attitudes as a function of norm, status dimension, status level, and participant age (with standard error bars).

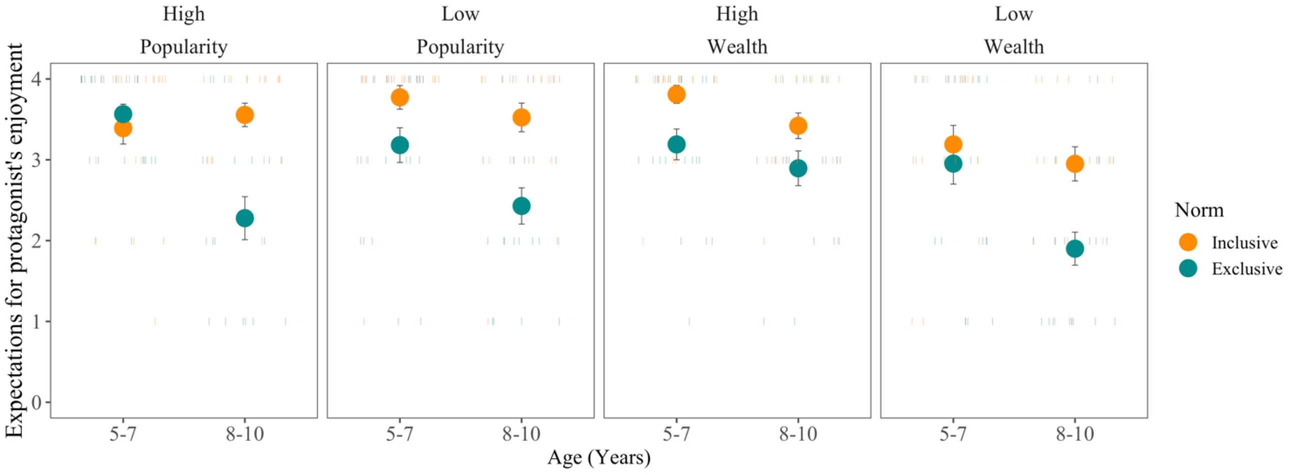

Supplementary Figure 2. Expectations for protagonist enjoyment as a function of norm, status dimension, status level, and participant age (with standard error bars).

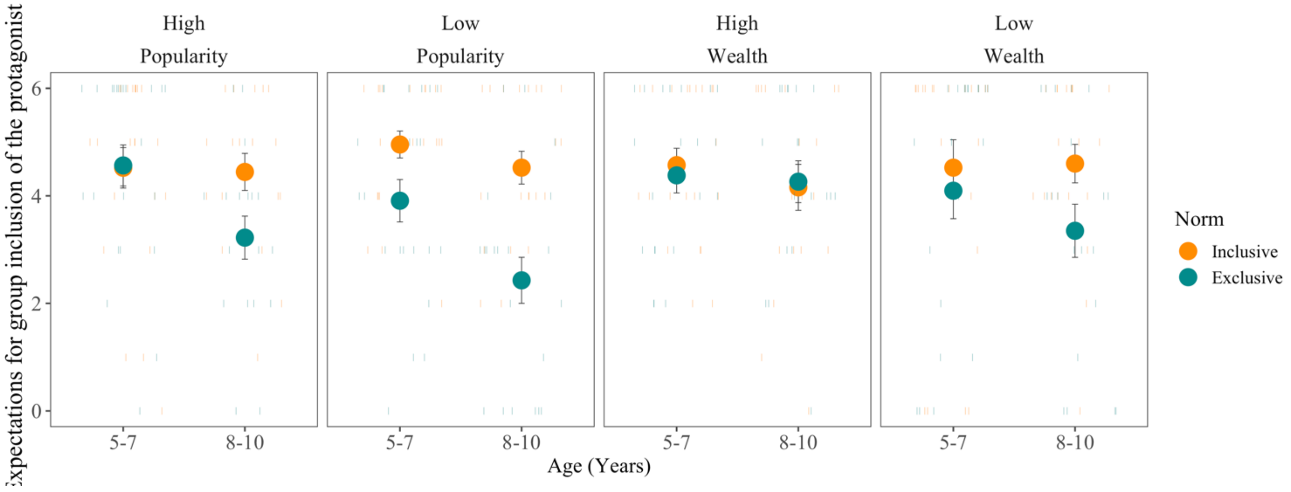

Supplementary Figure 3. Expectations for group inclusion of the protagonist as a function of norm, status dimension, status level, and participant age (with standard error bars).
